# Role of Faropenem in Treatment of Pediatric Infections: The Current State of Knowledge

**DOI:** 10.7759/cureus.24453

**Published:** 2022-04-25

**Authors:** Sumitha Nayak, Uday Pai, Amita Birla

**Affiliations:** 1 Department of Pediatrics, Shishu—The Children’s Clinic, Bangalore, IND; 2 Department of Pediatrics, Sai Kutir Clinic, Mumbai, IND; 3 Department of Clinical Research, Act Lifesciences Pvt Ltd, Navi Mumbai, IND

**Keywords:** methicillin-resistant staphylococcus aureus (mrsa), respiratory tract infections, children, carbapenems, β-lactamase, acute otitis media

## Abstract

Carbapenems play an important role in the management of bacterial infections. Meropenem, imipenem, ertapenem, and faropenem are carbapenems with the broadest antibacterial spectrum and strong antibacterial activity. Faropenem is a novel oral carbapenem with an advantage over other parenteral carbapenems in the series. Like other β-lactam antibiotics, faropenem inhibits cell wall synthesis by inhibiting penicillin-binding proteins (PBPs). Faropenem is stable against β-lactamase and has a low propensity for bacterial resistance. Faropenem has demonstrated excellent in-vitro and clinical activity in adult infections with a broad spectrum of activity. Faropenem also has a favorable safety profile. These activities of the faropenem created the interest of researchers in exploring its use in the treatment of pediatric infections. After promising outcomes in-vitro and clinical evaluation in children, faropenem is now approved in some parts of the world for the treatment of pediatric infections. Faropenem oral dry syrup is available for the treatment of a wide range of pediatric infections, including upper respiratory tract infections, urinary tract infections, dermatological infections, and bacterial periodontal infections in children. The current recommended clinical dose in pediatric patients is 15 mg/kg/ day, divided into three doses. The availability of faropenem dry syrup has expanded the current therapeutic options for treating pediatric infections.

In this review, we have put light on the in-vitro and clinical studies of faropenem dry syrup in pediatric patients, along with its molecular and pharmacological basics.

## Introduction and background

In children, bacterial infections are one of the most common infectious diseases. In nearly 40% of infections in children, co-infection with bacterial and viral pathogens is reported. Pediatricians frequently encounter respiratory tract infections (RTIs), mainly the upper RTIs like acute otitis media (AOM), sinusitis, and pharyngitis. Among children worldwide, acute respiratory infections (ARIs) are a primary cause of morbidity and a major cause of child mortality [1]. There is also a high incidence of pneumonia among children. *Streptococcus pneumoniae* is the most common bacterial cause of pneumonia [1]. AOM is the most common childhood infectious disease. By the age of three years, three in every four children will have ≥1 episode of AOM. A timely and accurate clinical diagnosis is required to provide optimal management in children with AOM. Knowing the course of the infection and the bacterial pathogens most frequently involved with a middle ear infection is also important during the management of AOM [1]. It is important to remember that respiratory pathogens isolated from children are less susceptible to many antibacterial agents than isolates from adults.

Urinary tract infections (UTIs) are common among children with a high incidence in infants and young children. By adolescent age, about 11% of girls and 7% of boys have had at least one episode of UTI, with recurrent infections in many cases. UTIs in children are generally managed as an outpatient; however, these are also among the most common cause of hospital admission in children [1]. Skin and soft-tissue infections (SSTIs) are becoming more common in children. Bacterial periodontal infections are reasons for visits to the pediatrician. Children are generally affected with a mild form of gingivitis [2]. Judicious use of antimicrobial agents is important in managing bacterial infections in children. Increasing antibiotic resistance makes it difficult to prescribe an empiric antibiotic treatment for pediatric infections. Current clinical evidence should guide antibiotic therapy in the pediatric setting [1,3].

This review focuses on different aspects of faropenem activity in children. To our knowledge, this is the first review article that compiles the evidence on faropenem in the pediatric setting.

## Review

Faropenem: a novel oral carbapenem

Carbapenems occupy a niche place in the antibiotic armamentarium. Among the range of different β-lactams, carbapenems possess the broadest spectrum of activity together with the greatest potency against Gram-positive and Gram-negative bacteria and aerobes responsible for a wide range of infectious diseases in children [4].

They are unique among the β-lactams currently available as they are relatively resistant to hydrolysis by most β-lactamases. Inhibition of β-lactamases is a “value-added feature” of carbapenem antibiotics [5]. Meropenem, imipenem, ertapenem, and faropenem are atypical β-lactam antibiotics having the broadest antibacterial spectrum together with strong antibacterial activity. They are important antibacterial drugs available to treat severe bacterial infections. Among these carbapenems, meropenem, imipenem, and ertapenem are strictly injectable antibiotics; these are not convenient for use in outpatient settings. Faropenem is a novel oral carbapenem that is now available for use in children [6].

Faropenem has a unique penem structure with structural similarity to penicillins and cephalosporins [4]. Faropenem is a prodrug that gets rapidly cleaved to release the microbiologically active faropenem following absorption into plasma. The activity of the faropenem is unaffected by β-lactamase-producing pathogens [4]. Faropenem is also resistant to Enterobacteriaceae organisms producing TEM-, SHV-, and CTX-M-type extended-spectrum β-lactamase (ESBL) [7]. Laboratory and longitudinal surveillance studies have reported that the faropenem has a low propensity for developing resistance [8]. Faropenem has demonstrated an excellent safety profile with a low incidence of diarrhea, nausea, or vomiting. There is also no identified risk of cardiotoxicity or seizures reported with this drug [9]. Collectively, all these properties of the faropenem make it a promising antibacterial to treat serious infections in children. Currently available as faropenem dry syrup for oral suspension, recommended in two dosage schedules, 15 mg/kg/day and 10 mg/kg/day in three divided doses.

Chemistry of faropenem

**Figure 1:**
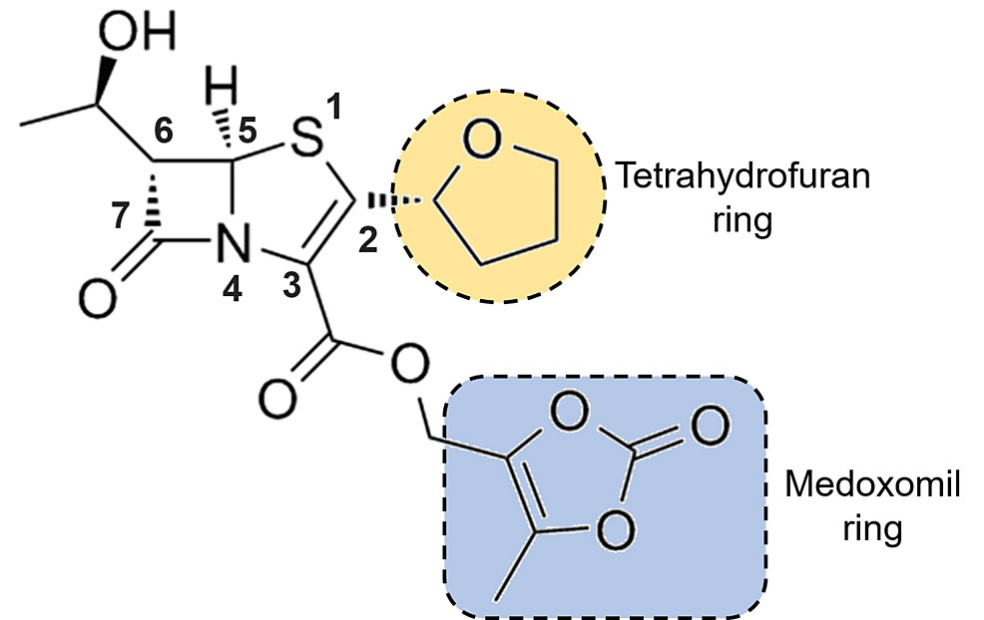
Structure of faropenem showing tetrahydrofuran and medoxomil ring and positions of caron atoms in penem structural core The figure is drawn by the author.

The penem structural core of the faropenem is an entirely synthetic molecule that has the 4:5 fused ring of a combination of penam and cephem nuclei. A four-membered β-lactam ring is fused to a 5-membered ring with a double bond between C-2 and C-3, but with the substitution of carbon for sulfur at C-1 [5] (Figure 1).

Like other β-lactam antibiotics, the inherent strain in the β-lactam ring provides a high degree of reactivity responsible for its antibacterial activity, which is affected by the nature of the ring to which it is conjugated. The double bond between C2 and C3 increases the reactivity of the β-lactam ring to nucleophiles, including the active site serine of penicillin-binding proteins (PBPs) [10]. The hydrogen atoms at C5 and C6 in faropenem structure are in the trans orientation with S stereochemistry at C6. The trans configuration of the β-lactam ring at C5 and C6 results in a high degree of stability against degradation by most β-lactamases [5,10].

Many structural features of the faropenem distinguish it from all β-lactams, including penicillins, cephalosporins, and other carbapenems. The position of sulfur in the faropenem differentiates it from the carbon-containing pyrrolidine ring of the other carbapenems [10]. The addition of a chiral tetrahydrofuran ringside chain at C2 of faropenem makes the critical differences in stability and activity compared with other carbapenems. The C2 side chain of the faropenem makes it stable to hydrolysis by the renal enzyme dehydropeptidase I (DHP-I), which has eliminated the need for co-administration with DHP inhibitors [10]. The formulations of the faropenem are mainly available in the form of ester prodrug form, where the medoxomil portion of the faropenem medoxomil is attached via an ester linkage to position C3 of the five-membered ring [5,10].

Mechanism of action of faropenem

**Figure 2:**
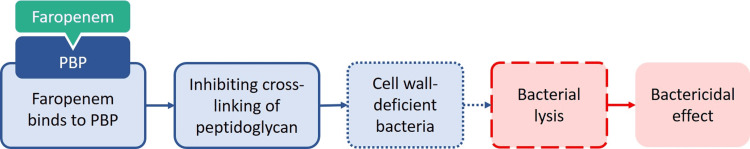
Mechanism of action of faropenem PBP: penicillin-binding protein The figure is drawn by the author.

Like other β-lactam antibiotics, faropenem exhibits bactericidal activity by inhibiting the cell wall synthesis via inhibition of PBPs. The β-lactam ring present in the faropenem mimics the D-alanine-D-alanine portion of the disaccharide peptide precursor. PBPs hydrolyze the β-lactam ring to form an intermediate that covalently binds to the serine residue of the active site inactivating the PBP [7,11]. Faropenem inhibits the cross-linking of peptidoglycan or transpeptidation, which further induces an antibacterial effect (Figure 2) [7,11].

Evaluation of the affinity of faropenem for several PBPs in *Staphylococcus aureus*, *Escherichia coli*, *Proteus vulgaris*, *Serratia marcescens*, *Pseudomonas aeruginosa*, *Enterococcus faecalis*, and *S. pneumoniae* has demonstrated that the drug has a higher affinity for the high-molecular-weight PBPs (PBP-1, -2, and -3) for most bacteria. Transpeptidases are the high-molecular-weight (>50 kD) PBPs [7,11-12]. Faropenem preferentially targets PBP2 and has a high affinity for PBP1A, PBP1B, and PBP3 [10].

Mechanism of resistance to faropenem

There are multiple mechanisms reported for the development of resistance to carbapenems. These include the production of β-lactamases, efflux pumps, and mutations that alter the expression and/or function of porins and PBPs. When more than one mechanism is present, there is a high resistance level to carbapenems in certain bacterial species, like *Klebsiella pneumoniae*, *P. aeruginosa*, and *Acinetobacter baumannii* [5]. Alteration of PBPs leads to decreased target binding affinity and the production of β-lactamases inactivating the drug [10].

Despite having a higher binding affinity for the high-molecular-weight PBPs than imipenem, *P. aeruginosa* is intrinsically resistant to faropenem due to poor outer membrane penetration [13]. Faropenem is refluxed by the multidrug efflux pump, MexAB-OprM; however, it appears to have a unique binding site and thus is not likely to select for efflux-mediated carbapenem resistance [13].

β-lactamase stability of faropenem

Faropenem has demonstrated intrinsic stability to β-lactamases in classes A, C, and D, including (extended-spectrum β-lactamases) ESBLs and AmpC β-lactamases [14]. The production of metallo β-lactamases, which are zinc-dependent class B β-lactamases, results in resistance to faropenem. However, the resistance to these β-lactamases was found to be five times lower with faropenem compared to imipenem. Also, the class B β-lactamases are currently rare [10,14]. Faropenem demonstrated overall good activity in susceptibility studies with a large collection of clinical isolates of a wide range of multidrug-resistant Gram-positive and Gram-negative bacterial species. However, methicillin-resistant *S. aureus* (MRSA), *Enterococcus faecium*, and Acinetobacter spp. were largely resistant to faropenem [6]. Faropenem is stable to penicillinase derived from *S. aureus* and *E. coli. *It is also highly stable against *E. coli*, and *P. vulgaris-*derived cephalosporinase. Faropenem is highly stable against hydrolysis by various β-lactamases prepared from *Bacteroides fragilis* strains [14].

In-vitro activities of faropenem against isolates from children

A study assessed antimicrobial susceptibilities of 393 *S. pneumoniae* isolates prospectively collected from children ≤14 years old [15]. Specimen sources for the isolates were non-invasive (sputum, bronchoalveolar lavage fluid, tracheal aspirate, ear, and sinus fluid), invasive (blood), or carriage (nasopharyngeal swab). Faropenem had the lowest MIC90 value of 1 μg/mL against the most prevalent serotype 19A isolates (Table 1) [15].

**Table 1:** Antimicrobial susceptibilities of the most prevalent 19A serotypes of Streptococcus pneumoniae isolated from children TMP-SMX: trimethoprim-sulfamethoxazole

Antimicrobials	MIC_90_	% of isolates		
		Susceptible	Intermediate	Resistant
Faropenem	1 μg/mL			
Amoxicillin/clavulanate	8 μg/mL	62	3	35
Cefdinir	>8 μg/mL	46	2	53
Cefuroxime axetil	16 μg/mL	46	2	52
Penicillin	4 μg/mL	13	36	50
Azithromycin	>16 μg/mL	36	0	64
TMP-SMX	>8 μg/mL	22	5	73
Telithromycin	1 μg/mL	99	1	0
Levofloxacin	1 μg/mL	100	0	0

Another study evaluated the activity of the faropenem against pathogens isolated from the middle ear fluid of children with AOM [8]. A total of 1,188 isolates were collected from children with AOM, of which 561 were *S. pneumoniae*, 554 were *Haemophilus influenzae,* 43 were *Moraxella catarrhalis*, and 30 were *Streptococcus pyogenes*. More than 90% of the isolates were collected from children of ≤2 years of age, and the remaining 10% were collected from children of ≤8 years of age. The susceptibility of isolates was tested against faropenem and nine other oral antimicrobials (ampicillin, amoxicillin-clavulanate, cefdinir, cefuroxime, penicillin, telithromycin, azithromycin levofloxacin, and trimethoprim-sulfamethoxazole (TMP-SMX)). Faropenem was the most active β-lactam against *S. pneumoniae* and *H. influenza*. It was also the most active β-lactam against the penicillin-resistant strain of *S. pneumoniae*. Faropenem also demonstrated activity against *M. catarrhalis* and *S. pyogenes* [8].

When the in-vitro activity of faropenem was compared with other β-lactam antibiotics, including co-amoxiclav, second-and third-generation cephalosporins, and clindamycin and metronidazole, it demonstrated a broad spectrum of activity and stability to group 2B-β-lactamases [4]. A total of 726 strains were studied of which four were NCTC control strains (*E. coli *10418, *P. aeruginosa* 10662, *S. aureus *6571, and *B. fragilis* 9343), 11 were well-characterized β-lactamase producers, and 711 were recent clinical isolates including Gram-negative, Gram-positive, and anaerobic bacteria. The activity of faropenem was compared with those of cefpodoxime, cefuroxime, cefixime, amoxicillin-clavulanate, benzylpenicillin, cefdinir, clindamycin, metronidazole, and cephaloridine. Faropenem had significant in-vitro activity against common respiratory pathogens, *H. influenzae*, *S. pneumoniae*, and *M. catarrhalis*. Faropenem had considerable activity against both methicillin-susceptible *S. aureus* (MSSA) and MRSA compared to amoxicillin-clavulanate and the cephalosporins. Compared to second-and third-generation cephalosporins, the faropenem was more stable to hydrolysis by the ESBLs. However, the faropenem showed poor in-vitro activity against *P. aeruginosa*, *Stenotrophomonas maltophilia*, and *E. faecium* [4].

Pharmacokinetics of faropenem

Faropenem is readily absorbed following the oral administration. The bioavailability of the faropenem falls between 72 and 84% in different studies [7,10]. The pharmacokinetic studies in children showed that the maximum plasma concentration (Cmax) is achieved in one hour after the oral administration of faropenem syrup [16]. Table 2 summarizes the key pharmacokinetic properties of faropenem dry syrup in children [7,10,17,18].

**Table 2:** Key pharmacokinetic properties of faropenem in children

Faropenem dose (mg/kg/dose)	Bioavailability	Maximum plasma concentration (C_max_)μg/mL	Time to achieve C_max _(T_max_) hr	Half-life (t_1/2_) hr	Urinary recovery rates %
5	72-84%	1.08 ± 0.38	~ 1 hr	2.72 ± 1.86	1.05-12.3
10		2.50 ± 1.81		1.14 ± 0.88	1.6-4.33

The values of Cmax and area under the curve (AUC) did not differ significantly when faropenem absorption was studied from different sections of the gastrointestinal tract [7]. These values did not differ when faropenem was administered under fasting and postprandial conditions. This suggests that faropenem can be administered without regard to food [7]. The preclinical and clinical studies evaluating the faropenem showed that it has approximately 90-95% protein-binding, which was also found to be saturable. The primary protein involved was human serum albumin [7,10,19]. Renal DHP-I hydrolyzes faropenem to its inactive metabolites, M-1 and M-2 which lack antimicrobial activity [7,10]. Nearly 8-26% of faropenem is eliminated unchanged in the urine. Active renal tubular secretion is the primary pathway for the elimination of the faropenem. The elimination half-life of the faropenem is 1-2 hours [7,10,20].

Pharmacodynamics of faropenem

The pharmacodynamic activity of the faropenem is dependent on both time and concentration. Bacterial killing by faropenem is time-dependent, and persistent effects, like the postantibiotic effect (PAE), are concentration-dependent [10,21]. Faropenem has proven bactericidal activity against clinical isolates of a wide range of organisms, including *B. fragilis*, *E. coli*, *K. pneumoniae*, *Proteus mirabilis*, *S. aureus*, *Staphylococcus* *saprophyticus*, *Streptococcus milleri*, *H. influenzae*, *M. catarrhalis*, and *S. pyogenes* [22,23]. Faropenem exhibited its fastest killing effect on *E. coli* and its slowest killing effect on *B. fragilis* [23].

The interaction between faropenem and serum proteins is complex. The clinical significance of high protein binding of faropenem is not understood completely. The presence of serum protein was associated with both an increase and decrease in the minimum inhibitory concentration (MIC) of faropenem in in-vitro studies. This activity was found to be dependent on the concentration of serum protein and also on the studied pathogen [7]. Faropenem exhibited a significant PAE against all control and clinical isolates of *S. aureus* and *S. pneumoniae* and all clinical isolates of *E. coli*. However, the duration of PAEs did not correlate with the bactericidal activity of the faropenem [7]. Indications of faropenem in pediatric patients are summarized in Table 3.

**Table 3:** Indications of faropenem in pediatric patients

Bronchitis	Cervical lymphadenitis
Bacterial pneumonia	Skin and soft-tissue infections
Pharyngitis	Cellulitis
Laryngitis	Vulvitis
Otitis media	Acute colitis
Tonsillitis	Purulent lymphadenitis
Sinusitis	Balanoposthitis
Pertussis (whooping cough)	Balanitis
Scarlet fever	Periodontitis
Urinary tract infections	

Dosage of faropenem in pediatric patients

Dose-ranging studies in children have suggested that faropenem at the dose of ≥15 mg/kg of body weight/day administered in divided doses achieves the pharmacokinetic concentration required to eradicate multidrug-resistant *S. pneumoniae* [15]. The current recommended clinical dose in pediatric patients is 15 mg/kg/day divided in three doses as 5 mg/kg/dose.

Clinical data in pediatric patients

Clinical Efficacy

The activity of faropenem in in-vitro studies is also reflected in clinical studies. Different clinical studies on children showed that faropenem is effective and safe in treating various bacterial infections in pediatric patients, including polymicrobial infections [24-28]. In these clinical studies, the use of faropenem dry syrup was associated with high patient acceptance, resulting in better compliance. Clinical studies evaluating faropenem in various infections with a broad range of microbial involvement have shown high efficacy rates (Table 4) [24-28] and high bacterial eradication rates (Table 5) [24-28].

**Table 4:** Clinical efficacy rates of faropenem

Infections	Clinical efficacy rates
Upper respiratory tract infection	90%
Bronchitis	100%
Otitis media	94.10%
Urinary tract infections	100%
Pharyngitis	50-100%
Tonsillitis	100%
Pneumonia	100%
Pertussis (whooping cough)	100%
Scarlet fever	100%
Impetigo contagiosa	33.33-85.71%
Cellulitis	100%
Balanoposthitis	100%
Balanitis	100%
Cervical lymphadenitis	100%
Staphylococcal scalded skin syndrome (SSSS)	100%
Vulvitis	100%
Acute colitis	100%
Purulent lymphadenitis	100%

**Table 5:** Bacteriological eradication rates of faropenem

Bacterial pathogen	Eradication rates
Staphylococcus aureus	100%
Streptococcus pneumoniae	75-100%
Streptococcus pyogenes	89-100%
Enterococcus faecalis	100%
Moraxella catarrhalis	82-100%
Escherichia coli	83-100%
Haemophilus influenzae	86-100%
Haemophilus parainfluenzae	50%
Proteus mirabilis	100%
Citrobacter freundii	0-100%

Yokota and co-workers evaluated faropenem in pediatric patients with bacterial infectious diseases [24]. A total of 112 pediatric patients of age ≤16 years were treated with oral faropenem dry syrup at the dose of 15-30 mg/kg/day divided over three doses. Overall treatment duration was 3-8 days. In the case of group A streptococcal infection, 5-14 days of treatment were given. Faropenem was found to be clinically effective in 91% of all patients. It also demonstrated a high bacteriological eradication rate with potent antibacterial activity [24]. In another study, 45 children with bacterial infection received oral faropenem at 3.4-10 mg/kg/dose three times a day for 3-14 days [25]. Excellent clinical responses were reported, with an overall efficacy rate of 93.3%. The overall bacteriological eradication rate of faropenem was 78.9% [25].

A study evaluating faropenem in children <10 years of age included 25 patients with different bacterial infections [26]. These patients received oral faropenem dry syrup at 18.5-20.5 mg/kg divided into three doses. The treatment duration was 4 to 11 days. Faropenem treatment resulted in an overall efficacy rate of 96% with an overall high bacteriological eradication rate of 93.3% [26]. Nishimura and co-workers evaluated oral faropenem dry syrup in pediatric patients (11 to 15 years of age) with bacterial infections [27]. Patients received faropenem at 18.5-20.5 mg/kg divided into three doses for 3 to 11 days. The overall efficacy rate was 96.8%, with an overall bacterial eradication rate of 81.0% [27].

Another study by Fujii and co-workers evaluated faropenem in polymicrobial infections [28]. A total of 494 pediatric patients diagnosed with polymicrobial infections received a mean dose of faropenem 4-11 mg/kg/dose thrice daily. Children were mainly diagnosed with mild to moderate pneumonia, acute bronchitis, pharyngitis (RTIs), cellulitis, impetigo (SSTIs), UTIs, and otitis media. Clinical efficacy rates were high with 88-100% efficacy rate in the case of polymicrobial infections. The bacteriological eradication rate was also found to be 82% in these cases [28].

Clinical safety and tolerability

The safety profile of the faropenem is excellent. During the clinical evaluation of the faropenem, no serious adverse events were reported. The only side effect reported was diarrhea; however, the incidence of diarrhea with faropenem syrup was similar to that with other oral β-lactam antibiotics [24-28].

## Conclusions

The efficacy rate of faropenem was found to be as high as 100%, with a favorable safety profile in clinical studies conducted on children. Faropenem dry syrup also enjoys a high acceptance rate by children resulting in better adherence to the treatment, ultimately resulting in better clinical outcomes. Faropenem should be used rationally in this set of populations and should be reserved for a later line of therapy.

These properties of faropenem make it a preferable antibiotic to be used in the pediatric setting. In light of the increased emergence of ESBL-producing strains of bacteria, faropenem may prove useful in the pediatric setting.
